# Recurrent Massive Epistaxis from an Anomalous Posterior Ethmoid Artery

**DOI:** 10.1155/2016/8504348

**Published:** 2016-11-29

**Authors:** Marco Giuseppe Greco, Francesco Mattioli, Maria Paola Alberici, Livio Presutti

**Affiliations:** Unità Operativa Complessa di Otorinolaringoiatria, Azienda Ospedaliero-Universitaria Policlinico di Modena, Italy Via del Pozzo 71, 41124 Modena, Italy

## Abstract

A 50-year-old man, with no previous history of epistaxis, was hospitalized at our facility for left recurrent posterior epistaxis. The patient underwent surgical treatment three times and only the operator's experience and radiological support (cranial angiography) allowed us to control the epistaxis and stop the bleeding. The difficult bleeding management and control was attributed to an abnormal course of the left posterior ethmoidal artery. When bleeding seems to come from the roof of the nasal cavity, it is important to identify the ethmoid arteries always bearing in mind the possible existence of anomalous courses.

## 1. Introduction

Epistaxis is the most common emergency encountered by the otolaryngologist-head and neck surgeon and affects all age groups, although with different incidence rates. It is most common before age 10 and between ages 45 and 65 years [1, 2].

Nasal packing usually provides good control of epistaxis but sometimes, especially in arterial bleeding, surgical treatment represents the only available treatment and can be particularly troublesome, even for experienced surgeons [3].

We report an unusual case of epistaxis arising from the left posterior ethmoid artery, which presented an abnormal course.

Control of epistaxis was particularly difficult and was only achieved after three surgical interventions.

## 2. Case Report

We report the case of a 50-year-old man affected by hypertension and chronic ischemic heart disease with no previous history of epistaxis and who we hospitalized at our facility for left recurrent posterior epistaxis.

During the 6 days before hospitalization, the patient had attended the emergency room of our otolaryngology department on a number of occasions for recurrent left epistaxis: two times for left nasal packing with two Merocel® Standard dressings; once for nasal packing with one Merocel Standard dressing in the right fossa and one Rapid Rhino® + Tabotamp® in the left fossa. On the following day, after further bleeding, he was hospitalized after nasal packing with one Merocel Standard dressing in the right fossa and a Bivona® Silicone Epistaxis Catheter in the left fossa.

During the previous epistaxis, we noted a major source of bleeding that seemed to come from the roof of the nasal fossa. Another significant element was the onset of bleeding: it was sudden and violent but discontinuous. The patient did not present any coagulation disorders and blood pressure had always remained in the normal range.

On the following day, the patient first underwent cerebral and maxillofacial computed tomography (CT) with normal results. Angio-CT of the carotid and cerebral circulation did not reveal intracranial vascular malformations or the presence of vascular aneurysms.

Meanwhile, as a result of the violent nose bleeding, the patient's hemoglobin level continued to fall until it reached 8.9 g/dL; for this reason, he underwent surgery. We removed the nasal swabs previously placed. From nasal endoscopy, we noticed the presence of widespread mucosal bleeding from the roof of the left nasal cavity but could not identify the source of bleeding. We aspirated clots and cauterized the inferior turbinate. We then removed the head of the middle turbinate to better visualize the back of the nasal cavity. We performed middle meatal antrostomy, identification of the posterior wall of the maxillary sinus, and identification of the sphenopalatine artery and its cauterization. Anterior ethmoidectomy with nasal packing was then carried out. There was no bleeding upon awakening.

The next day, further surgical treatment was required for left recurrent epistaxis. Under endoscopic control, the surgeon removed the nasal swabs and several washes were performed to remove clots. Left posterior ethmoidectomy was completed. The arterial bleeding seemed to originate from the ethmoid roof. The anterior ethmoid artery was isolated from its bony shell so as to perform accurate cauterization of the artery. At the end of the procedure, there was no further epistaxis and blood pressure was normal (120/80 mmHg).

On the following day, a new violent left epistaxis occurred which resolved spontaneously after a few minutes. Given the context, we planned urgent angiography to exclude the presence of vascular malformations, abnormal arterial courses, or serious injury to the internal carotid artery in the sphenoid sinus.

**Figure 1:**
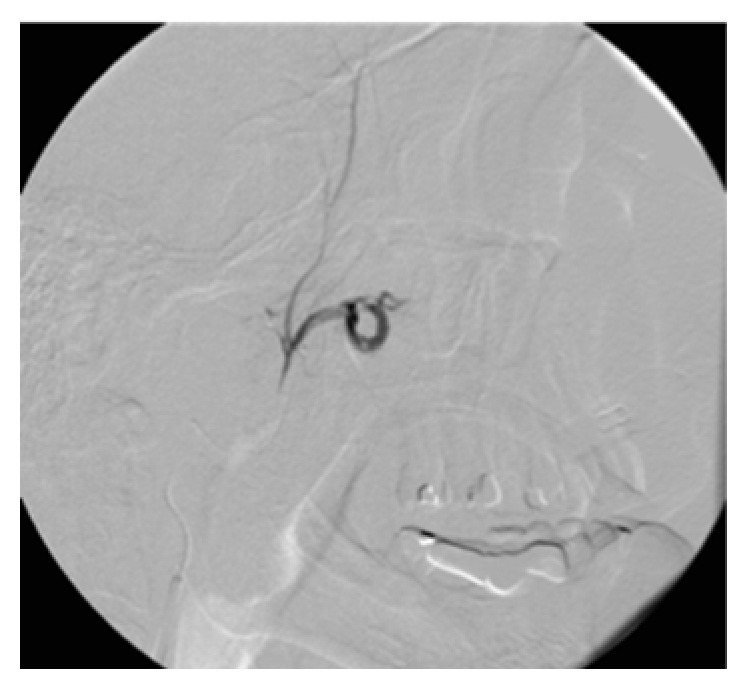
Left internal maxillary artery embolization.

**Figure 2:**
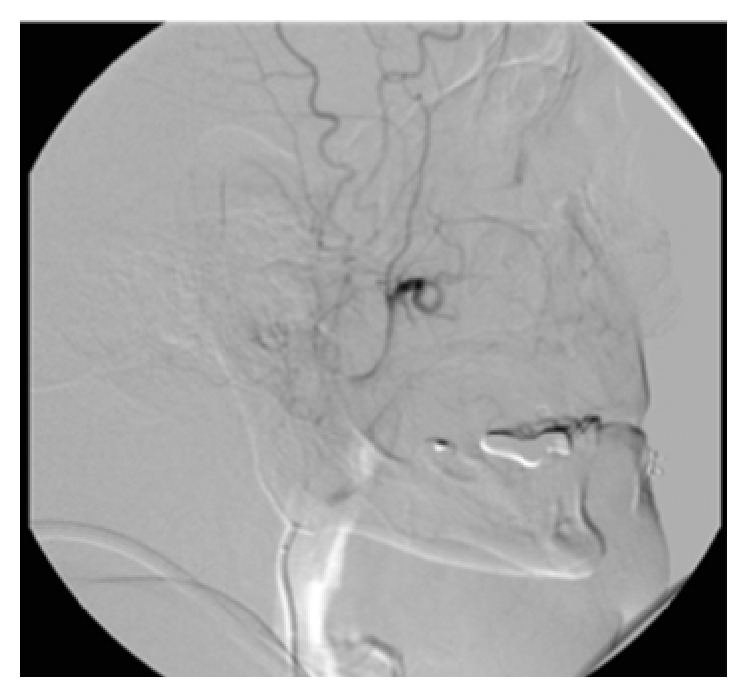
Persistent nosebleeds after embolization.

**Figure 3:**
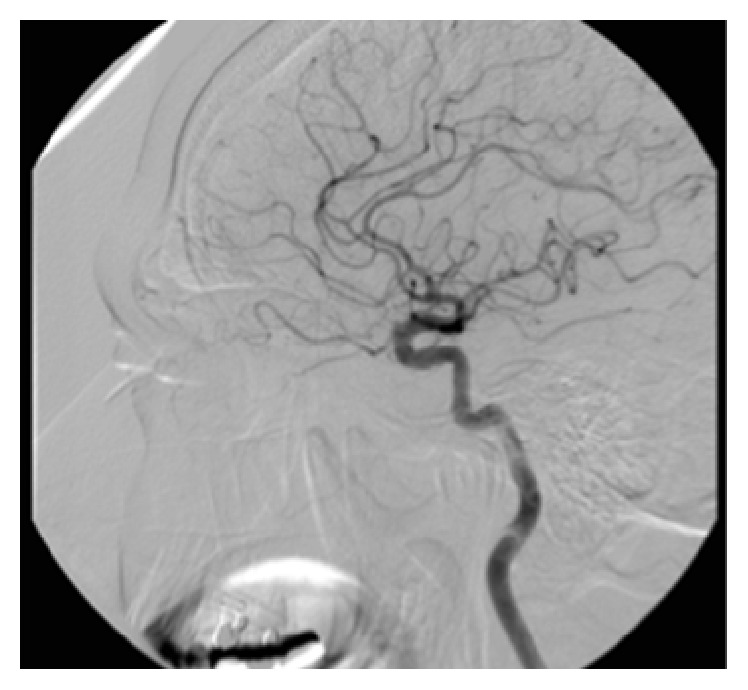
Ethmoid vascularization by branches from the left ophthalmic artery.

The exam was performed 2 days later by femoral selective catheterization of the internal and external carotid arteries bilaterally. The onset of epistaxis was noted during the exam, and so the radiologist performed embolization of both internal maxillary arteries (Figures 1 and 2) and the left facial artery with injection of polyvinyl alcohol particles (500 *μ*m) but did not achieve control of the bleeding. Selective catheterization of the internal carotid artery bilaterally showed significant ethmoid vascularization by branches from the ophthalmic artery (anterior and posterior ethmoid arteries) with small irregularities of the branches (Figure 3).

**Figure 4:**
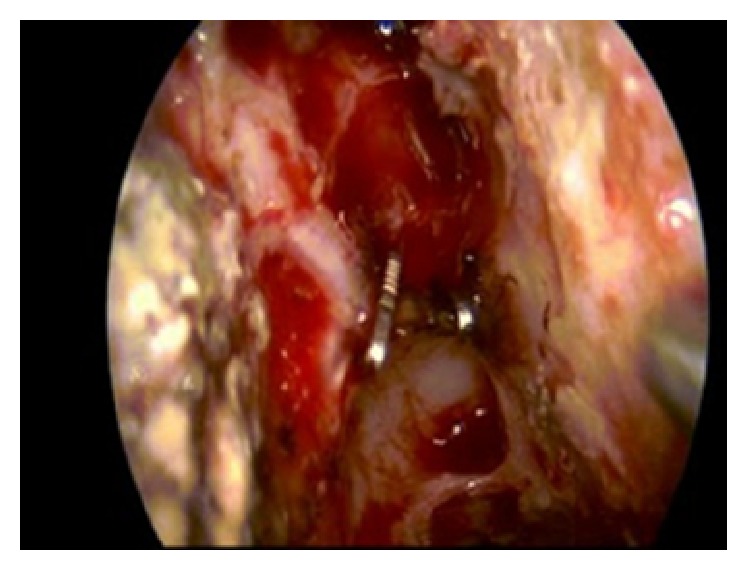
Endoscopic image: anterior ethmoid artery: closure with metal clips.

**Figure 5:**
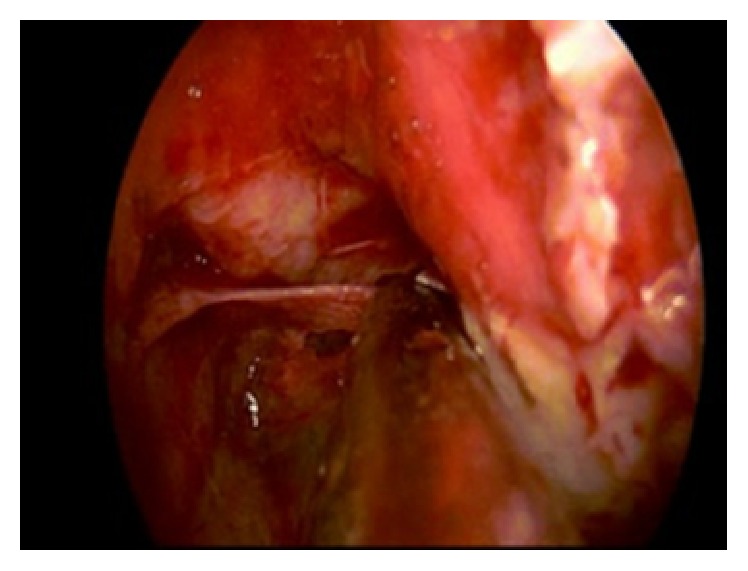
Endoscopic image: posterior ethmoidal artery in its bony shell.

**Figure 6:**
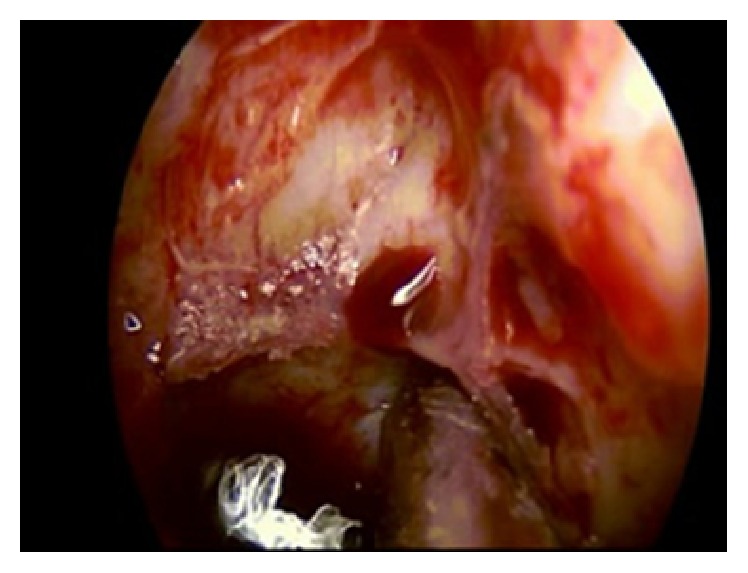
Endoscopic image: posterior ethmoidal artery between the optic nerve and the carotid canal.

**Figure 7:**
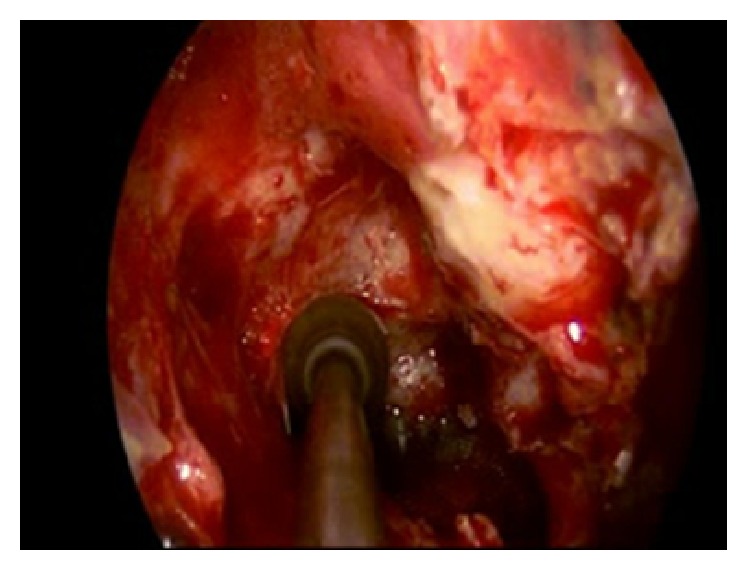
Endoscopic image: posterior ethmoidal artery: hemostasis with diamond drill.

**Figure 8:**
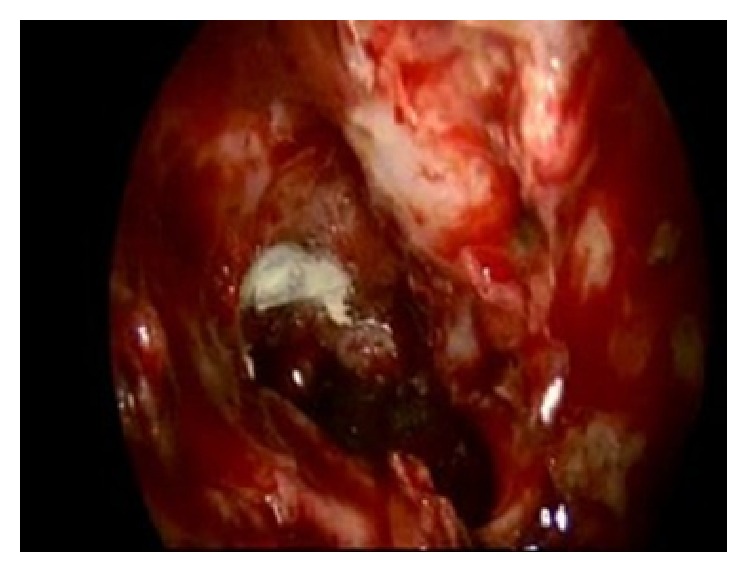
Endoscopic image: posterior ethmoidal artery: hemostasis with diamond drill.

On the same day, the patient underwent surgery a further time. Also on this occasion, bleeding seemed to originate from the ethmoid roof. During this last revision surgery we found the remains of the anterior ethmoid artery cauterized above the roof of the ethmoid; we closed the anterior ethmoid artery with clips (Figure 4), then, using an endoscope, we explored the sphenoid sinus. Here we found that the posterior ethmoid artery presented an abnormal path, located between the canal of the optic nerve and the carotid canal (Figures 5 and 6); at this level, there was a small dehiscence responsible for the bleeding. The artery was covered by a bony shell along its intrasinual course which made thermal cauterization with bipolar forceps impractical so the artery was cauterized using a diamond drill and this achieved control of the bleeding (Figures 7 and 8).

## 3. Discussion

Posterior bleeding (10%) usually originates from the sphenopalatine artery (terminal branch of the internal maxillary artery) or from its branches or, more rarely, from the anterior or posterior ethmoid arteries, branches of the ophthalmic artery [4, 5].

The posterior ethmoidal artery (PEA) originates from the ophthalmic artery, but in the posterior third of the orbit. Many anatomical variations frequently occur at the origin of the PEA (86%) [6, 7]. The PEA can also originate from the third part of the ophthalmic artery (5%), or from the second part of the ophthalmic artery (5%). At its origin, it runs between the rectus superior and the superior oblique muscle and then emerges from the myofascial cone of the orbit to finally pass perpendicular to the medial wall and enter the posterior ethmoidal canal (PEC); the PEA runs toward the medial orbital wall, crossing obliquely from the upper part of the superior oblique muscle and trochlear nerve [8]. On its intraorbital course, it follows a superior convex loop and ends up above the oblique muscle. The PEA has a small caliber, usually less than 1 mm, which makes its identification difficult in CT studies; the intraorbital part has a 0.66 ± 0.21 mm diameter and the intracranial part has a 0.45 ± 0.12 mm (range, 0.32 to 0.57 mm) diameter. In the study by Tomkinson et al. [1, 2], they could identify the PEA in only 14/40 nasal fossae.

Then PEA crosses the roof of the ethmoid labyrinth inside the homonymous canal 2-3 mm from the anterior wall of the sphenoid sinus. PEA into its canal presents a more horizontal orientation than that of the AEA with an entry angle into the skull base of between 0° and 18° [9]. During nasal surgery, it is very important to remember that the PEA runs very close to the optic nerve: the distance between the two structures is variable and can range from 4 to 16 mm; for this reason, extreme care is required during approaches that require working in this territory. A damage to the artery may cause hemorrhages resulting in a surgical eye emergency in cases where an orbital hematoma forms rapidly as a result of retraction of the lacerated artery into the orbit.

PEA is usually identified in its bony canal at the insertion of the partitioning roof of the middle turbinate but in our patient it presented an abnormal path: we found it into the lateral wall of the sphenoid sinus located between the canal of the optic nerve and the carotid canal; at this level, there was a small dehiscence responsible for the bleeding.

Surgical experience is very important especially in the management of complex cases of posterior epistaxis.

Radiological examinations represent an important aid but only if surgeons require specific and useful investigations: in our patient, angio-CT did not contribute to resolution of the case; on the contrary, it slowed the diagnostic process and subsequent surgical resolution.

On the contrary, the anatomical findings of the radiologist during angiography allowed the surgeon to focus his attention on the branches of the ophthalmic artery (anterior and posterior ethmoid arteries) and to identify the origin of the bleeding.

We must also emphasize that, in addition to its indisputable diagnostic value, angiography is the only radiological investigation that may be used for therapeutic purposes through selective catheterization and embolization of the bleeding vessel.

Finally, it is worth remembering the role of clinical observation. In most cases, arterial bleeding originates from the sphenopalatine artery or its branches, and its cauterization solves the problem. When bleeding seems to come from the roof of the nasal cavity, it is important to identify the ethmoid arteries always bearing in mind the possible existence of anomalous courses.

## References

[1] Tomkinson A., Roblin D. G., Flanagan P. (1997). Patterns of hospital attendance with epistaxis. *Rhinology*.

[2] Nikoyan L., Matthews S. (2012). Epistaxis and hemostatic devices. *Oral and Maxillofacial Surgery Clinics of North America*.

[3] Douglas R., Wormald P.-J. (2007). Update on epistaxis. *Current Opinion in Otolaryngology and Head and Neck Surgery*.

[4] Supriya M., Shakeel M., Veitch D., Ah-See K. W. (2010). Epistaxis: prospective evaluation of bleeding site and its impact on patient outcome. *Journal of Laryngology and Otology*.

[5] McClurg S. W., Carrau R. L. (2014). Endoscopic management of posterior epistaxis: a review. *Acta Otorhinolaryngologica Italica*.

[6] Lang J., Schäfer K. (1979). Arteriae ethmoidales: ursprung, verlauf versorgungsgebiete und anastomosen. *Cells Tissues Organs*.

[7] Monjas-Cánovas I., García-Garrigós E., Arenas-Jiménez J. J., Abarca-Olivas J., Sánchez-Del Campo F., Gras-Albert J. R. (2011). Radiological anatomy of the ethmoidal arteries: CT cadaver study. *Acta Otorrinolaringologica Espanola*.

[8] Erdogmus S., Govsa F. (2006). The anatomic landmarks of ethmoidal arteries for the surgical approaches. *Journal of Craniofacial Surgery*.

[9] Han J. K., Becker S. S., Bomeli S. R., Gross C. W. (2008). Endoscopic localization of the anterior and posterior ethmoid arteries. *Annals of Otology, Rhinology and Laryngology*.

